# Tamoxifen for prevention of breast cancer: extended long-term follow-up of the IBIS-I breast cancer prevention trial

**DOI:** 10.1016/S1470-2045(14)71171-4

**Published:** 2015-01

**Authors:** Jack Cuzick, Ivana Sestak, Simon Cawthorn, Hisham Hamed, Kaija Holli, Anthony Howell, John F Forbes, on behalf of the IBIS-I Investigators

**Affiliations:** a Centre for Cancer Prevention, Wolfson Institute of Preventive Medicine, Queen Mary University London, London, UK; b Breast Care Centre, Southmead Hospital, Bristol, UK; c Guy's and St Thomas Hospital, London, UK; d The University of Tampere, Pirkanmaa Cancer Society, Tampere, Finland; e Genesis Breast Cancer Prevention Centre, Manchester, UK; f University of Newcastle, Calvary Mater Hospital, Australia New Zealand Breast Cancer Trials Group Newcastle, Australia

## Abstract

**Background:**

Four previously published randomised clinical trials have shown that tamoxifen can reduce the risk of breast cancer in healthy women at increased risk of breast cancer in the first 10 years of follow-up. We report the long-term follow-up of the IBIS-I trial, in which the participants and investigators remain largely masked to treatment allocation.

**Methods:**

In the IBIS-I randomised controlled trial, premenopausal and postmenopausal women 35–70 years of age deemed to be at an increased risk of developing breast cancer were randomly assigned (1:1) to receive oral tamoxifen 20 mg daily or matching placebo for 5 years. Patients were randomly assigned to the two treatment groups by telephone or fax according to a block randomisation schedule (permuted block sizes of six or ten). Patients and investigators were masked to treatment assignment by use of central randomisation and coded drug supply. The primary endpoint was the occurrence of breast cancer (invasive breast cancer and ductal carcinoma in situ), analysed by intention to treat. Cox proportional hazard models were used to assess breast cancer occurrence and mortality. The trial is closed to recruitment and active treatment is completed, but long-term follow-up is ongoing. This trial is registered with controlledtrials.com, number ISRCTN91879928.

**Findings:**

Between April 14, 1992, and March 30, 2001, 7154 eligible women recruited from genetics clinics and breast care clinics in eight countries were enrolled into the IBIS-I trial and were randomly allocated to the two treatment groups: 3579 to tamoxifen and 3575 to placebo. After a median follow up of 16·0 years (IQR 14·1–17·6), 601 breast cancers have been reported (251 [7·0%] in 3579 patients in the tamoxifen group *vs* 350 [9·8%] in 3575 women in the placebo group; hazard ratio [HR] 0·71 [95% CI 0·60–0·83], p<0·0001). The risk of developing breast cancer was similar between years 0–10 (226 [6·3%] in 3575 women in the placebo group *vs* 163 [4·6%] in 3579 women in the tamoxifen group; hazard ratio [HR] 0·72 [95% CI 0·59–0·88], p=0·001) and after 10 years (124 [3·8%] in 3295 women *vs* 88 [2·6%] in 3343, respectively; HR 0·69 [0·53–0·91], p=0·009). The greatest reduction in risk was seen in invasive oestrogen receptor-positive breast cancer (HR 0·66 [95% CI 0·54–0·81], p<0·0001) and ductal carcinoma in situ (0·65 [0·43–1·00], p=0·05), but no effect was noted for invasive oestrogen receptor-negative breast cancer (HR 1·05 [95% CI 0·71–1·57], p=0·8).

**Interpretation:**

These results show that tamoxifen offers a very long period of protection after treatment cessation, and thus substantially improves the benefit-to-harm ratio of the drug for breast cancer prevention.

**Funding:**

Cancer Research UK (UK) and the National Health and Medical Research Council (Australia).

## Introduction

Breast cancer remains the most common type of cancer in women, with an estimated incidence of 1·6 million cases per year worldwide.^1^ Tamoxifen is a well-established and effective treatment for oestrogen receptor-positive breast cancer.^2^ Four large randomised clinical trials have shown that tamoxifen reduces the incidence of oestrogen receptor-positive breast cancer in women at high risk of developing the disease.^3^, ^4^, ^5^, ^6^, ^7^, ^8^, ^9^ A recently published meta-analysis of all prevention trials investigating selective oestrogen receptor modulators has shown that these drugs significantly reduce the incidence of all breast cancer (including ductal carcinoma) in the first 10 years of follow-up (hazard ratio [HR] 0·62 [95% CI 0·56–0·69]).^10^ The HR for tamoxifen was 0·67 (95% CI 0·59–0·76), but this was maintained for the entire 10-year period (HR 0·62 [95% CI 0·53–0·73] in years 0–5 and 0·78 [0·62–0·97] in years 5–10), whereas little follow-up information was available after 5 years for the other selective oestrogen receptor modulators.^10^

The International Breast cancer Intervention Study I (IBIS-I) was initiated in 1992 and recruited women at high risk of developing breast cancer to receive oral tamoxifen (20 mg daily) or matching placebo. The initial report showed a significant reduction (odds ratio [OR] 0·68 [95% CI 0·50–0·92]) for all types of breast cancer (including ductal carcinoma in situ) after a median follow-up of 4·2 years (IQR 2·67–5·58).^6^ After a median follow-up of 8 years (IQR 6·35–9·61), an updated report showed the significant reduction for all types of invasive breast cancer continued (risk ratio 0·73 [95% CI 0·58–0·91]) with tamoxifen.^7^ In both reports, a risk reduction by tamoxifen was only seen for oestrogen receptor-positive breast cancer and ductal carcinoma in situ. As has been reported elsewhere,^5^, ^9^, ^11^, ^12^ thromboembolic and gynaecological adverse events were increased with tamoxifen compared with placebo during active treatment, but the 8-year update showed that those side-effects were mainly confined to the active treatment period.^5^, ^7^

With the exception of the Royal Marsden trial, in which median follow-up was 13 years,^5^ follow-up has been limited to 10 years or less for all other reports. In this Article, we report an updated analysis of the IBIS-I trial, which remains largely masked to treatment allocation.

## Methods

### Study design and participants

For this randomised controlled trial, eligible women 35–70 years of age from 37 centres (genetics clinics and breast care clinics) in eight countries (the UK, Australia, New Zealand, Finland, Spain, Switzerland, Belgium, and Ireland; appendix p 2) and judged to be at increased risk of developing breast cancer were enrolled and randomly assigned in a 1:1 ratio to receive oral tamoxifen 20 mg daily or matching placebo for 5 years (appendix p 3). Patients were deemed to be at an increased risk of developing breast cancer based on a family history of breast cancer or abnormal benign breast disease. Specific details about eligibility, entry, and exclusion criteria have previously been described in full.^6^, ^7^ In brief, women had to have risk factors for breast cancer indicating at least a twofold increased risk for the disease in women aged 45–70 years, whereas this risk needed to be higher than twofold for those younger than 45 years of age. Women with a history of any invasive cancer (excluding skin cancer), deep vein thrombosis, pulmonary embolism, or who wanted to become pregnant were excluded from trial participation. Women were defined as postmenopausal if they had 12 consecutive months of amenorrhea or had an oophorectomy. Menopausal hormone therapy use was allowed during the trial. All participants provided written informed consent, after an initial discussion with their IBIS-I doctor and a consideration period of at least 24 h. The trial was approved by local ethics committees for each participating centre.

### Procedures

Women were actively followed up for 5 years in the clinic or by telephone at 6-monthly intervals. All women have completed active treatment and are being followed up for occurrence of breast cancer, any other cancer, major adverse events, and death. In the UK, cancers and deaths are also reported to the IBIS-I central office by the Office for National Statistics. Adverse events were collected by annual postal questionnaires, which were sent directly to all participants and returned to the central office. In the non-UK centres, annual questionnaires, annual clinic visits, or hospital notes were used to collect these data, supplemented by a national registry in Finland. Treatment allocation still remains largely masked for investigators and participating women who have not developed breast or any other cancer. An option was given to women who had not developed breast cancer after a minimum of 10 years' follow-up on IBIS-I to take additional preventive therapy through enrolment into the IBIS-II trial.^13^

### Randomisation and masking

Randomisation was done centrally by the IBIS study group (with no external input) by telephone or fax to a central office in London, UK, for the European centres and in Sydney, Australia, for the centres in Australia and New Zealand. Balanced block randomisation was used and stratified by centre. Initial blocks of eight were created and were then randomly permuted into blocks of six or ten, to ensure that the final entry in each block was not predictable. The non-consecutive allocation sequence was generated by the IBIS-I programmer before study commencement. All IBIS-I personnel, participants, and clinicians were masked to treatment allocation and only the IBIS-I trial statistician (IS) had access to unmasked data.

### Outcomes

The primary endpoint was the occurrence of any type of breast cancer (including ductal carcinoma in situ). Secondary endpoints included the occurrence of invasive oestrogen receptor-positive breast cancer, all-cause mortality, and adverse events.

### Statistical analysis

The cutoff date for this analysis was May 1, 2014, and events that occurred after this date were not included. All information received before Oct 1, 2014, was included in our analyses. Only major adverse events, such as death, other cancers, thromboembolic events and cardiovascular events, are reported in this analysis. All analyses were by intention to treat, and efficacy endpoints were based on HRs from Cox proportional hazard models^14^, ^15^ with corresponding 95% CIs. We checked proportionality using Schoenfeld residuals^16^ and we estimated survival curves using the Kaplan-Meier method.^17^ Women who joined the IBIS-II trial and were randomly allocated to active treatment (anastrozole) were censored at that point. We compared secondary endpoints using logistic regression. We used Fisher's exact tests to compare adverse events between the tamoxifen and placebo groups when appropriate. All p values were two-sided. We used STATA version 12.1 for all analyses.

This study is registered with controlled-trials.com, number ISRCTN91879928.

### Role of the funding source

The funders had no role in study design, collection of data or material, data analysis, interpretation of the data, or writing of the report. JC and IS had full access to all the raw data. The corresponding author had access to all the data and had final responsibility for the decision to submit for publication.

## Results

Between April 14, 1992, and March 30, 2001, 7169 women were initially enrolled into the trial and randomly assigned to the two treatment groups. 15 women were subsequently found to be ineligible because of previous breast cancer diagnosis (nine of those assigned to placebo and six assigned to tamoxifen), leaving a total of 7154 women in the trial (3579 in the tamoxifen group and 3575 in the placebo group). Median follow-up for this analysis was 16·0 years (IQR 14·1–17·6), and a total of 110 043 women-years of follow-up have been accrued (tamoxifen: 55 419, placebo: 54 624). Most women (6639 [93%] of 7154) have had more than 10 years of follow-up, and the cumulative number of women-years of follow-up are 69 074 before 10 years and 40 969 thereafter. Median age at study entry was 49·9 years (IQR 46·0–55·0), and 3858 (54%) of 7154 women were postmenopausal. 4002 (56%) of 7154 had a body-mass index (BMI) higher than 25 kg/cm^2^, and 2876 (40%) of 7154 used menopausal hormone therapy at some point during the active treatment phase of the trial. Use of this treatment during the trial was slightly, but not significantly, higher in women assigned to tamoxifen.^7^ Appendix p 3 shows other baseline demographics. Most women (6939 [97%] of 7154) were entered into the trial because of a family history of breast cancer, but a few (572 [8%]) were enrolled on the basis of having a benign breast lesion associated with increased breast cancer risk.

At the time of data cutoff, treatment allocation still remains largely masked for investigators and participating women who have not developed breast or any other cancer (2702 [75·5%] of those assigned to tamoxifen *vs* 2646 [74·0%] of those who received placebo). After 10 years' follow-up, 603 women joined the IBIS-II trial, of whom 302 were randomly assigned to anastrozole and were censored at that time.

**Figure 1:**
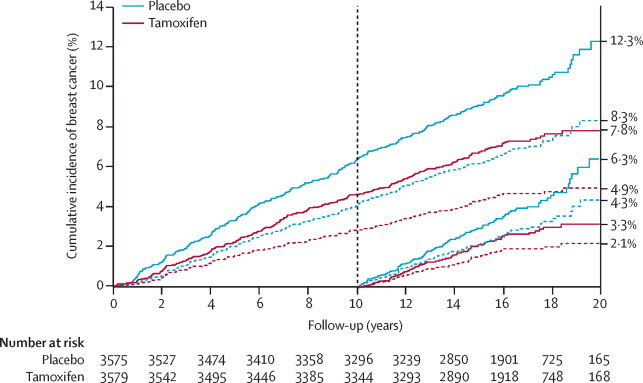
Cumulative incidence of breast cancers over time All breast cancers (solid lines) and invasive oestrogen receptor-positive breast cancers (dashed lines), according to treatment group and duration of follow-up.

**Figure 2:**
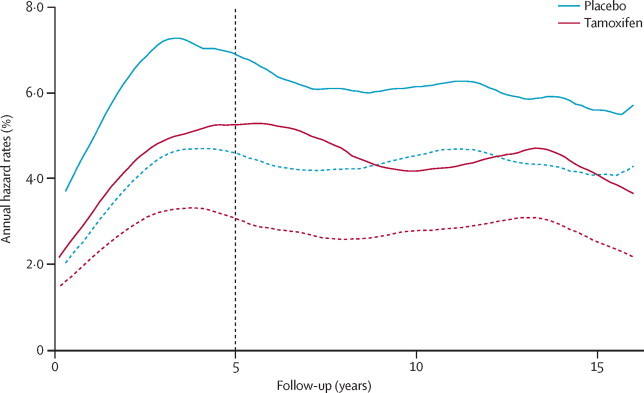
Smoothed annual hazard rate curves for breast cancer All breast cancers (solid lines) and invasive oestrogen receptor-positive breast cancers (dashed lines), according to treatment group.

A total of 601 breast cancers were reported before the cutoff date of May 1, 2014 (251 [7·0%] in 3579 women in the tamoxifen group *vs* 350 [9·8%] of 3575 in the placebo group; table 1). We found a significant reduction in the occurrence of all breast cancers in the tamoxifen group compared to the placebo group (HR 0·71 [95% CI 0·60–0·83], p<0·0001). We observed a significant reduction in the first 10 years of follow-up (HR 0·72 [95% CI 0·59–0·88], p=0·001), which was slightly greater in subsequent years (0·69 [0·53–0·91], p=0·009). Figure 1 shows the Kaplan-Meier-based cumulative incidence curves for all breast cancers (and for oestrogen receptor-positive invasive cancers) according to follow-up period. After 20 years of follow-up, the estimated risk of developing all types of breast cancer was 12·3% (95% CI 10·1–14·5) in the placebo group compared with 7·8% (95% CI 6·9–9·0) in the tamoxifen group, indicating that the number needed to treat for 5 years to prevent one breast cancer in the next 20 years was 22 (95% CI 19–26). Figure 2 shows the hazard rates by year for tamoxifen and placebo and confirms the continuing benefit of tamoxifen versus placebo over the entire 20-year follow-up period. A test for proportionality of hazards indicated no departure from the proportional hazards assumption during the entire follow up period (p=0·9).

**Table 1:** Breast cancers according to treatment allocation, cancer characteristics, and follow-up period

		**0–10 year follow-up period**				**≥10 year follow-up period**				**Overall**				**Ratio of ≥10 years:0–10 years**	
		Placebo (n=3575)	Tamoxifen (n=3579)	HR (95% CI)	p value	Placebo (n=3295)	Tamoxifen (n=3343)	HR (95% CI)	p value	Placebo (n=3575)	Tamoxifen (n=3579)	HR (95% CI)	p value	Ratio (95% CI)	p value
Women-years of follow-up		34 411	34 663	..	..	20 213	20 756	..	..	54 624	55 419	..	..	..	..
Total number of breast cancers		226	163	0·72 (0·59–0·88)	p=0·0011	124	88	0·69 (0·53–0·91)	p=0·0075	350	251	0·71 (0·60–0·83)	p<0·0001	0·98 (0·74–1·30)	p=0·91
Age (years)															
	≤50	100	65	0·65 (0·47–0·88)	p=0·0054	61	36	0·58 (0·3–0·87)	p=0·0074	161	101	0·62 (0·48–0·79)	p<0·0001	0·91 (0·58–1·39)	p=0·65
	>50	126	98	0·77 (0·59–1·01)	p=0·055	63	52	0·80 (0·56–1·16)	p=0·24	189	150	0·78 (0·63–0·97)	p=0·025	1·06 (0·72–1·56)	p=0·75
Menopausal hormone therapy use															
	Never/formerly	143	87	0·60 (0·46–0·79)	p=0·0002	82	54	0·64 (0·45–0·90)	p=0·011	225	141	0·62 (0·50–0·76)	p<0·0001	1·08 (0·75–1·54)	p=0·65
	During study	83	76	0·91 (0·67–1·24)	p=0·55	41	34	0·81 (0·52–1·27)	p=0·36	124	110	0·88 (0·68–1·13)	p=0·31	0·91 (0·56–1·46)	p=0·67
Ductal carcinoma in situ		38	21	0·55 (0·32–0·93)	p=0·027	15	14	0·91 (0·44–1·89)	p=0·81	53	35	0·65 (0·43–1·00)	p=0·047	1·69 (0·76–3·75)	p=0·17
Invasive cancer		188	141	0·74 (0·60–0·93)	p=0·0078	101	73	0·70 (0·52–0·95)	p=0·022	289	214	0·73 (0·61–0·87)	p<0·0001	0·96 (0·70–1·32)	p=0·81
Oestrogen receptor-positive cancer*		145	100	0·68 (0·53–0·88)	p=0·0033	93	60	0·63 (0·45–0·87)	p=0·0044	238	160	0·66 (0·54–0·81)	p<0·0001	0·94 (0·66–1·31)	p=0·69
Oestrogen receptor-negative cancer*		43	40	0·92 (0·60–1·42)	p=0·72	4	10	2·43 (0·76–7·75)	p=0·13	47	50	1·05 (0·71–1·57)	p=0·79	2·69 (0·76–11·74)	p=0·09
Grade*															
	Low	35	28	0·79 (0·48–1·31)	p=0·36	19	14	0·72 (0·36–1·43)	p=0·35	54	42	0·77 (0·51–1·15)	p=0·19	0·92 (0·43–1·94)	p=0·82
	Intermediate	90	59	0·65 (0·47–0·90)	p=0·0095	68	42	0·60 (0·41–0·88)	p=0·0085	158	101	0·63 (0·49–0·81)	p<0·0001	0·94 (0·63–1·40)	p=0·77
	High	62	52	0·83 (0·58–1·20)	p=0·33	13	17	1·27 (0·62–2·62)	p=0·52	75	69	0·91 (0·66–1·26)	p=0·57	1·56 (0·71–3·49)	p=0·23
Nodal status*															
	Negative	128	92	0·71 (0·55–0·93)	p=0·014	62	45	0·71 (0·48–1·04)	p=0·077	190	137	0·71 (0·57–0·89)	p=0·0022	1·01 (0·67–1·51)	p=0·96
	Positive	54	43	0·79 (0·53–1·18)	p=0·25	33	25	0·73 (0·44–1·24)	p=0·25	87	68	0·77 (0·56–1·06)	p=0·11	0·95 (0·54–1·65)	p=0·86
Tumour size*															
	≤1 cm	56	41	0·73 (0·49–1·09)	p=0·12	26	20	0·75 (0·42–1·34)	p=0·33	82	61	0·73 (0·53–1·02)	p=0·067	1·05 (0·56–1·96)	p=0·86
	1–2 cm	84	52	0·61 (0·44–0·87)	p=0·0051	39	28	0·70 (0·43–1·14)	p=0·15	123	80	0·64 (0·49–0·85)	p=0·0018	1·16 (0·69–1·93)	p=0·55
	>2 cm	48	48	0·99 (0·67–1·48)	p=0·97	36	25	0·68 (0·41–1·13)	p=0·13	84	73	0·86 (0·63–1·17)	p=0·33	0·69 (0·40–1·19)	p=0·16

Data are n or HR (95% CI), unless otherwise indicated. p values are for treatment differences, on 1 degree of freedom unless otherwise indicated. p values for heterogeneity or trend (all on 1 degree of freedom) are as follows: age=0·2, menopausal hormone therapy use=0·04, oestrogen receptor status=0·04, grade=0·38, nodal status=0·6, tumour size=0·94. HR=hazard ratio.

* For invasive breast cancer only.

A similar pattern was observed for invasive oestrogen receptor-positive breast cancer (table 1, figure 1). A significant reduction in cancer occurrence with tamoxifen was recorded in the first 10 years of follow-up, which was maintained in subsequent years (table 1, figure 2). The number needed to treat to prevent one invasive oestrogen receptor-positive breast cancer was 29 (95% CI 26–34).

**Figure 3:**
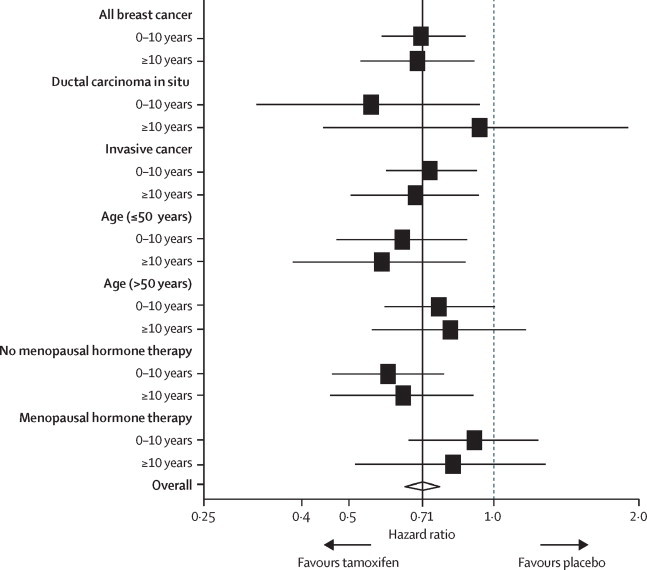
Forest plot for subgroup analyses according to follow-up periods (0–10 years *vs* ≥10 years) Horizontal lines are 95% CIs.

A significant reduction for tamoxifen was also recorded for ductal carcinoma in situ, but only in the first 10 years of follow-up (table 1). No significant effect with tamoxifen was recorded for invasive oestrogen receptor-negative breast cancer (table 1, figure 3). There were more oestrogen receptor-negative breast cancers in the tamoxifen group after 10 years of follow-up than in the placebo group (ten cancers in the tamoxifen group *vs* four in the placebo group; HR 2·45 [0·77–7·82], p=0·13). The preventive effects of tamoxifen did not differ according to tumour size, nodal status, or grade, since there was substantial overlap in the confidence intervals and no significant trends (table 1, appendix p 4).

Women who had menopausal hormone therapy during the 5 years of active treatment had significantly less benefit from tamoxifen than those who did not (p=0·04; table 1, figure 3). This effect was larger for women who developed invasive oestrogen receptor-positive cancers (users of menopausal hormone therapy HR 0·87 [95% CI 0·64–1·19] *vs* non users 0·55 [0·42–0·72]; p=0·03). There was no significant difference between women aged 50 years or younger than in older women throughout the follow-up periods (table 1, figure 3). No interactions were recorded with other demographic factors (appendix p 4).

In total, 666 cancers other than breast cancer were reported (table 2). 315 (8%) other cancers occurred in women in the placebo group compared with 351 (9%) in the tamoxifen group (OR 1·13 [0·96–1·32], p=0·3) (table 2). Although not significant, there were more endometrial cancers in the tamoxifen group than the placebo group, but the excess was confined to the first 5 years of active treatment, with no subsequent difference (table 2). Other gynaecological cancers were distributed similarly between the two treatment groups (table 2). Significantly fewer gastrointestinal cancers occurred in women receiving tamoxifen than in those receiving placebo (42 in the tamoxifen group *vs* 63 in the placebo group; OR 0·66 [95% CI 0·44–0·99], p=0·038; table 2). Non-melanoma skin cancers were significantly increased in the tamoxifen group, whereas there was a similar incidence of melanoma skin cancers between the two treatment groups (table 2). More cases of lung cancer were reported with tamoxifen (32 cases) than with placebo (24 cases), although this difference was not significant and was only observed in the first 10 years of follow-up (table 2). No specific treatment or time-period differences were recorded for other cancers (table 2).

**Table 2:** Incidences of other types of cancers according to follow-up period and treatment allocation

		**0–5 year follow-up period**				**5–10 year follow-up period**				**≥10 year follow-up period**				**Overall**			
		Placebo (n=3575)	Tamoxifen (n=3579)	OR (95% CI)	p value	Placebo (n=3474)	Tamoxifen (n=3446)	OR (95% CI)	p value	Placebo (n=3295)	Tamoxifen (n=3343)	OR (95% CI)	p value	Placebo (n=3575)	Tamoxifen (n=3579)	OR (95% CI)	p value
Women-years of follow-up		17 558	17 648	..	..	16 852	17 015	..	..	20 213	20 756	..	..	54 624	55 419	..	..
Gynaecological cancers																	
	Endometrial	4	15	3·76 (1·20–15·56)	p=0·011	11	7	0·64 (0·21–1·80)	p=0·35	5	7	1·40 (0·38–5·61)	p=0·56	20	29	1·45 (0·79–2·71)	p=0·19
	Other	10	10	1·00 (0·37–2·68)	p=0·99	5	11	2·20 (0·70–8·09)	p=0·13	8	7	0·88 (0·27–2·77)	p=0·79	23	28	1·22 (0·68–2·22)	p=0·48
Gastrointestinal cancers																	
	Colorectal	11	13	1·18 (0·49–2·92)	p=0·68	12	9	0·75 (0·28–1·94)	p=0·51	24	13	0·54 (0·25–1·10)	p=0·069	47	35	0·74 (0·46–1·18)	p=0·18
	Other	4	2	0·50 (0·05–3·49)	p=0·41	5	2	0·40 (0·04–2·44)	p=0·26	7	3	0·43 (0·07–1·87)	p=0·20	16	7	0·44 (0·15–1·12)	p=0·059
Lung cancer		1	7	7·00 (0·90–315·69)	p=0·033	6	10	1·67 (0·55–5·59)	p=0·32	17	15	0·88 (0·41–1·88)	p=0·73	24	32	1·33 (0·76–2·37)	p=0·29
Skin cancers																	
	Melanoma	8	11	1·37 (0·50–3·94)	p=0·49	10	7	0·70 (0·23–2·04)	p=0·47	11	10	0·91 (0·35–2·36)	p=0·83	29	28	0·96 (0·55–1·68)	p=0·89
	Non-melanoma	24	20	0·83 (0·43–1·57)	p=0·54	16	30	1·88 (1·00–3·70)	p=0·039	44	66	1·51 (1·01–2·27)	p=0·034	84	116	1·39 (1·04–1·87)	p=0·022
Other cancers		16	9	0·56 (0·22–1·34)	p=0·16	12	15	1·25 (0·54–2·92)	p=0·57	14	16	1·14 (0·52–2·53)	p=0·72	42	40	0·95 (0·60–1·51)	p=0·82
	Lymphoma, myeloma, or leukaemia	6	9	1·50 (0·48–5·12)	p=0·44	5	6	1·20 (0·30–4·98)	p=0·76	8	15	1·88 (0·75–5·12)	p=0·14	19	30	1·58 (0·86–2·98)	p=0·12
	Brain	1	2	2·00 (0·10–117·9)	p=0·46	4	2	0·50 (0·05–3·49)	p=0·41	6	2	0·33 (0·03–1·84)	p=0·15	11	6	0·54 (0·17–1·61)	p=0·22
All cancers		85	98	1·16 (0·85–1·57)	p=0·33	86	99	1·16 (0·86–1·57)	p=0·33	144	154	1·08 (0·85–1·37)	p=0·52	315	351	1·13 (0·96–1·32)	p=0·15

OR=odds ratio. To calculate odds ratios, the number of women at risk at the beginning of the time interval is used as the denominator. To calculate relative risks, number of women-years of follow-up is used to compute rates.

Further data about minor side-effects were not recorded since the last publication^7^ because no effects were anticipated to occur more than 5 years after completion of treatment. However, information about major thromboembolic, cerebrovascular, and cardiac events continued to be collected (appendix p 3). At last data cutoff (May, 2014), there was a significantly higher incidence of deep vein thrombosis in women receiving tamoxifen than those receiving placebo (50 [1·4%] of 3579 women receiving tamoxifen *vs* 29 [0·8%] of 3575 women receiving placebo; OR 1·73 [95% CI 1·07–2·85], p=0·02; appendix p 3). However, the increased risk was only during the first 10 years of follow-up (46 [1·3%] in the tamoxifen group *vs* 25 [0·7%] in the placebo group; OR 1·87 [95% CI 1·11–3·18], p=0·011). No significant differences between treatment groups were recorded for major cardiovascular events (13 [<1%] of 3579 women in the tamoxifen group *vs* 17 [<1%] of 3575 women in the placebo group; OR 0·76 [95% CI 0·34–1·67], p=0·46) or cerebrovascular accidents (30 [1%] tamoxifen *vs* 28 [1%] placebo; 1·07 [0·62–1·86], p=0·80).

A total of 348 deaths were reported up until the cutoff date (182 [5·1%] of 3579 women in the tamoxifen group and 166 [4·6%] of 3575 women in the placebo group; table 3). There was no significant difference in mortality between groups (OR 1·10 [95% CI 0·88–1·37], p=0·4). The higher number of deaths in the tamoxifen group were confined to the first 10 years of follow-up (86 deaths in the tamoxifen group *vs* 71 in the placebo group), and a similar number of deaths were reported thereafter (96 tamoxifen *vs* 95 placebo). Table 3 shows the specific causes of deaths. Overall, tamoxifen had no effect on breast cancer-specific mortality (31 deaths with tamoxifen *vs* 26 with placebo; OR 1·19 [95% CI 0·68–2·10], p=0·8). A few more breast cancer deaths occurred in the tamoxifen group after 10 years of follow-up, although this difference was not significant (18 tamoxifen *vs* nine placebo; OR 2·00 [0·85–5·06], p=0·08). There was no evidence to suggest that these late deaths were associated with use of menopausal hormone therapy during the trial (six tamoxifen *vs* three placebo; p=0·33) or the development of oestrogen receptor-negative tumours (four tamoxifen *vs* two placebo; p=0·42). Five women in the tamoxifen group died from endometrial cancers (four within the first 10 years) compared with none in the placebo group (p=0·06). We recorded no significant differences in other cancers or causes of death, although there was a non-significant increase in respiratory deaths occurred in the tamoxifen group after 10 years (13 tamoxifen *vs* six placebo; OR 2·17 [95% CI 0·77–6·96], p=0·1).

**Table 3:** Specific causes of death according to treatment group and follow-up period

		**0–10 year follow-up period**		**≥10 year follow-up period**		**Overall**	
		Placebo (n=71)	Tamoxifen (n=86)	Placebo (n=95)	Tamoxifen (n=96)	Placebo (n=166)	Tamoxifen (n=182)
Cancer							
	Breast	17 (24%)	13 (15%)	9 (9%)	18 (19%)	26 (16%)	31 (17%)
	Genitourinary	7 (10%)	11 (13%)	9 (9%)	10 (10%)	16 (10%)	21 (12%)
	Digestive	8 (11%)	10 (12%)	12 (13%)	11 (11%)	20 (12%)	21 (12%)
	Lung	9 (13%)	9 (10%)	17 (18%)	9 (9%)	26 (16%)	18 (10%)
	Lymphatic	2 (3%)	2 (2%)	0	2 (2%)	2 (1%)	4 (2%)
	Melanoma	0	1 (1%)	1 (1%)	4 (4%)	1 (1%)	5 (3%)
	Other	7 (10%)	11 (13%)	6 (6%)	8 (8%)	13 (8%)	19 (10%)
Cardiac		3 (4%)	10 (12%)	11 (12%)	2 (2%)	14 (8%)	12 (7%)
Deep vein thrombosis or pulmonary embolism		2 (3%)	3 (4%)	1 (1%)	1 (1%)	3 (2%)	4 (2%)
Stroke or cerebrovascular accident		5 (7%)	4 (5%)	7 (7%)	6 (6%)	12 (7%)	10 (5%)
Infection		0	1 (1%)	3 (3%)	1 (1%)	3 (2%)	2 (1%)
Neurological		1 (1%)	0	1 (1%)	3 (3%)	2 (1%)	3 (2%)
Respiratory		2 (3%)	2 (2%)	6 (6%)	13 (14%)	8 (5%)	15 (8%)
Other		8 (11%)	8 (9%)	10 (11%)	7 (7%)	18 (11%)	15 (8%)
Unknown		0	1 (1%)	2 (2%)	1 (1%)	2 (1%)	2 (1%)

Percentages are proportions of all deaths for given treatment and time period.

## Discussion

This extended analysis of the IBIS-I trial provides important evidence showing that 5 years of tamoxifen treatment reduces the incidence of breast cancer for at least 20 years. In our previous report after an 8-year median follow-up,^7^ 337 breast cancers were reported. In the current report with 16-year median follow-up, the number of breast cancers has almost doubled to 601, of which 212 (35%) have occurred after 10 years of follow-up. The preventive effect of tamoxifen has remained similar throughout this 20-year period (panel). Reductions were recorded for invasive oestrogen receptor-positive cancers and ductal carcinoma in situ, but not for invasive oestrogen receptor-negative cancers. Of interest was a small, non-significant increase in oestrogen receptor-negative tumours after 10 years (ten cases with tamoxifen *vs* four with placebo). An increase in these tumours was also seen in the selective oestrogen receptor modulator meta-analysis but only in years 5–10,^10^ which was significant for all selective oestrogen receptor modulators (p=0·02), but not for tamoxifen alone (p=0·4). However, no follow-up was available after 10 years. This finding could be due to tumours that would have presented earlier as oestrogen receptor-positive cancers, but were transiently held in check by tamoxifen, and then later escaped the need for hormonal stimulus and subsequently appeared as oestrogen receptor-negative cancers. Follow-up beyond 10 years is limited to the Royal Marsden trial^5^ in which treatment was given for 8 years and median follow up was 13 years. Unlike other trials, the Royal Marsden trial reported little effect of tamoxifen in the 8 years of treatment (risk ratio 1·06 [95% CI 0·70–1·70], p=0·8), but a reduction in invasive cancers after 8 years (38 cancers *vs* 56 cancers; HR 0·67 [95% CI 0·44–1·01), p=0·05), with larger effects for oestrogen receptor-positive than for oestrogen receptor-negative cancers. The reason for a difference between early and late effects is unclear, but that trial randomly assigned a group of women who were younger and at higher risk of breast cancer than those in our trial, which could be relevant. Nevertheless, the agreement between these two trials regarding the long-term effectiveness of tamoxifen is important and provides strong evidence for a long-lasting reduction in number of cases of breast cancer.

Panel **Research in context**

**Systematic review**

A systematic review of the use of tamoxifen and other selective oestrogen receptor modulators for breast cancer prevention has recently been published,^10^ but contained follow-up for only 10 years. This review included the four randomised trials (including the present IBIS-I trial) that have reported about the use of tamoxifen for prevention of breast cancer.^3^, ^4^, ^5^, ^6^, ^7^, ^9^, ^11^, ^12^ We identified one only trial (the Royal Marsden trial) with follow-up beyond 10 years,^5^ but the number of breast cancers after 8 years in this trial was only 94, compared with 212 after 10 years for IBIS-I in our report.

**Interpretation**

The results from the present trial provide important additional evidence that 5 years of tamoxifen treatment reduces the incidence of breast cancer in high-risk women for the entire follow-up period. Effect sizes for tamoxifen were significantly greater in women who did not take menopausal hormone therapy during the treatment period—a finding that has not been reported previously. No effect on all-cause or breast cancer mortality was recorded, but an increase in deaths from endometrial cancer was confirmed as noted in the adjuvant trials.^18^ Our results substantially improve the benefit-to-harm ratio for the use of tamoxifen to prevent breast cancer in high-risk women, with an estimated 22 women being needed to be treated for 5 years to prevent one breast cancer in the next 20 years.

The benefit of tamoxifen was significantly greater in women who did not use menopausal hormone therapy during the treatment period than in those who used this therapy, indicating the clear loss of efficacy of tamoxifen when menopausal hormone therapy is used concomitantly. This finding was not reported in the Royal Marsden trial^5^, ^9^ or in the Italian trial (the Italian Tamoxifen Prevention Study),^11^, ^12^ and menopausal hormone therapy use was not allowed in the NSABP-P1 trial.^3^, ^4^ Understanding how the type and duration of menopausal hormone therapy affects breast cancer risk is thus an important question that has to be addressed. Full details are being abstracted (in a manuscript currently being prepared by the IBIS-I team) and will be presented elsewhere. Of interest was the large effect of tamoxifen on the incidence of ductal carcinoma in situ in the first 10 years and little effect thereafter. In the overview of selective oestrogen receptor modulators,^10^ the effect of tamoxifen and other selective oestrogen receptor modulators for ductal carcinoma in situ was largely restricted to the first 5 years, with little effect in years 5–10, but no data are available after 10 years. The relevance of this for breast cancer prevention is not clear, but since ductal carcinoma in situ is a precursor lesion, it could indicate that the preventive effect of tamoxifen will not be maintained indefinitely.

All women have completed the active follow-up portion of this trial, but continue to be monitored by a range of methods. In the UK and Finland, where 4412 (61·7%) of the participants were recruited, follow-up is covered by national flagging systems and so will be essentially complete for cancer and death. These data were augmented by annual questionnaires in the UK and clinic notes in Finland. For Australia and New Zealand (2676 [37·4%] of cases), annual questionnaires were used for all participants except for 44 women who have withdrawn consent for additional follow-up (and these women were censored at that time). Additionally, for this analysis we obtained up-to-date clinical follow up for the remaining few cases in Switzerland and Belgium (63 [0·9%] of cases). Long-term follow-up was not undertaken in Spain and Ireland (three [<0·1%] cases). Thus, we believe that the data for breast cancer and deaths are virtually complete. Data about other major but non-fatal side-effects (eg, strokes and myocardial infarctions) are substantially complete but are not covered by national flagging systems and so are only obtained from clinic visits, note review, and postal questionnaires. Data for minor side-effects (eg, vasomotor symptoms or gynaecological events) were reported after 8 years' median follow-up,^7^ and since these were mostly confined to the active treatment period they have not been collected beyond 10 years of follow-up.

Overall there was no significant difference in all-cause mortality between treatment groups, and the excess of death in the tamoxifen group was smaller than in previous reports.^6^, ^7^ The number of deaths was very similar in the two groups after 10 years. Despite there being no significant difference in the number of deaths from breast cancer between treatment groups, there were more deaths from breast cancer in the tamoxifen group; this outcome could not be linked to use of menopausal hormone therapy or the occurrence of oestrogen receptor-negative breast cancer. Endometrial cancer was more common in the tamoxifen group than in the placebo group during active treatment, but similar thereafter. However, deaths from endometrial cancer were more frequent in the tamoxifen group (five) than in the placebo group (none), and all but one occurred after 5 years of active treatment. Excess deaths from endometrial cancer have also been reported in the overview of adjuvant tamoxifen trials,^18^ with an annual rate slightly higher than reported here (1·9 *vs* 1·1 per 10 000 women-years), which at least partly represents the higher number of postmenopausal women in the adjuvant studies. These rates do not adjust for women who had undergone a hysterectomy, so the risk in women with a uterus would be about 50% higher than those reported here in IBIS-I. The apparent, although not statistically significant, excess of deaths from respiratory causes has not been reported in the overview of tamoxifen adjuvant trials, with only two respiratory deaths recorded in the 15-year follow-up^18^ (Pan H, University of Oxford, UK, personal communication), so this finding might well be due to chance alone.

The fact that the reduced incidence of breast cancer with tamoxifen has not translated into a mortality reduction is of some concern. However, the ratio of deaths from breast cancer (57) to incident cases of breast cancer (601) is only 9·5%, so the power for analysis of mortality (which our study was not powered to assess) is much lower than that for incidence. Additionally, the effect on mortality is expected to be lower than that for incidence since only oestrogen receptor-positive cancers are prevented by tamoxifen. We previously estimated that the recorded reduction in incidence would lead to an 18% reduction in breast cancer mortality.^19^ If this were the case, the power to detect such a reduction at this stage (with two-sided 5% significance level) would be only 12%, so the absence of a significant difference is not surprising.

In conclusion, our results clearly show a long-term effect of 5 years of tamoxifen treatment to prevent breast cancer in the next 20 years. No new late toxicity has been identified, although the early excess of endometrial cancer in the tamoxifen group has translated into an increased number of deaths, which, although not significant in our study, has been reported in adjuvant trials of tamoxifen and therefore is important in making the decision to use the drug. These results substantially improve the benefit-to-harm ratio for the use of tamoxifen to prevent breast cancer in high-risk women.
