# Supplementary material for: Tamoxifen for prevention of breast cancer: extended long-term follow-up of the IBIS-I breast cancer prevention trial
Source: Lancet Oncol. 2015 Jan;16(1):67–75. doi: 10.1016/S1470-2045(14)71171-4 (PMC4772450; doi:10.1016/S1470-2045(14)71171-4)

THE LANCET Oncology

Supplementary appendix

This appendix formed part of the original submission and has been peer reviewed. We post it as supplied by the authors.

Supplement to: Cuzick J, Sestak I, Cawthorn S, et al, on behalf of the IBIS-I Investigators. Tamoxifen for prevention of breast cancer: extended long-term follow-up of the IBIS-I breast cancer prevention trial. *Lancet Oncol* 2014; published online Dec 11. http://dx.doi.org/10.1016/S1470-2045(14)71171-4.

Webappendix

IBIS-I Working Party and Principal Investigators— E Abdi (Tweed Hospital, Tweed Heads, Australia); E Anderson (Western General Hospital, Edinburgh, UK); C Atkinson (St George's Cancer Care Centre, Christchurch, New Zealand); M Baum (University College London, London, UK; Clinical Adviser); J Beith (Royal Prince Alfred Hospital, Sydney, Australia); A Bird (Moorfields Hospital, London, UK; Ophthalmology Adviser); S Birrell (Flinders Medical Centre, Adelaide, Australia); R Blamey (Nottingham City Hospital, Nottingham, UK; deceased); R Blum (Bendigo Hospital, Bendigo, Australia); J Boyages (Westmead Hospital, Sydney, Australia); K Buser (Engeriedspital & Swiss Group for Clinical Cancer Research, Bern, Switzerland); I Campbell (Waikato Hospital, Hamilton, New Zealand); S Cawthorn (Southmead Hospital, Bristol, UK; Clinical Adviser); C Chapman (Oxford Radcliffe Hospital, Oxford, UK); M Chipman (Victorian Breast & Oncology Care, East Melbourne, Australia); A Coates (Royal Prince Alfred Hospital, Sydney, Australia; Clinical Adviser); J P Collins (Royal Melbourne Hospital, Melbourne, Australia); P Craft (Canberra Hospital, Canberra, Australia); J Cuzick (Queen Mary University London, London, UK; Chairman); L Denton (Glenfield Hospital NHS Trust, Leicester, UK); J Dewar (Sir Charles Gairdner Hospital, Nedlands, Australia); M Dowsett (Royal Marsden Hospital, London, UK); H Earl (Addenbrooke's Hospital, Cambridge, UK); D Eccles (University of Southampton, Southampton, UK); R Edwards (Queen Mary University London, London, UK;); G Evans (Christie Hospital NHS Trust, Manchester, UK); L Fallowfield (University of Sussex, Brighton, UK); I Fentiman (London Bridge Hospital, London, UK); J F Forbes (ANZ BCTG, University of Newcastle, Newcastle Mater Hospital, Australia; Clinical Adviser); M Friedlander (Prince of Wales Hospital, Sydney, Australia); J Garcia (Hospiat Universitario "Principe de Asturias", Madrid, Spain); W George (Western Infirmary, Glasgow, UK); F J Gilbert (University of Aberdeen, Aberdeen, UK); P Godbolt (Wesley Breast Clinic, Brisbane, Australia); A Goldhirsch (European Institute of Oncology, Milan, Italy); H Hamed (Guy's Hospital, London, UK; Clinical Adviser); A Hanby (St James's University Hospital, Leeds, UK; Trial Pathologist); S Hart (Monash Medical Centre, Clayton, Australia); J Hearne (Cancer Research UK, London, UK; Observer); A Henry (Hopital Jolimont, St Paul, Belgium); C Hirst (Wesley Breast Clinic, Brisbane, Australia); C Holcombe (Royal Liverpool University Hospital, Liverpool, UK); K Holli (Tampere University, and University Hospital, Finland); A Howell (Manchester Breast Centre, University of Manchester, Manchester, UK; Clinical Adviser); J Kirk (Westmead Hospital, Sydney, Australia); M Lansdown (St James's University Hospital, Leeds, UK); K Law (Cancer Research UK, London, UK; Observer); M Lee (City Hospital NHS Trust, Birmingham, UK); T Lennard (University of Newcastle upon Tyne, Newcastle Upon Tyne, UK); F MacNeil (London Breast Clinic, London, UK); P Maddox (Royal United Hospital, Bath, UK); R Mansel (University Hospital of Wales, Cardiff, UK); P McAleese (Beaumont Hospital, Dublin, Ireland); J MacKay (Addenbrooke's NHS Trust, Cambridge, UK); K MacMichael (Huddersfield Royal Infirmary, Huddersfield, UK); C Mitine (Hopital Jolimont, St Paul, Belgium); C Normand (London School of Hygiene and Tropical Medicine, London, UK); W Odling-Smee (Queen's University Belfast, Belfast, UK); T Oivanen (Pirkanmaa Cancer Society, Tampere, Finland); O Pagani (Ospedale Regionale della Beata Vergine, Mendrisio, Switzerland); T Powles (Cancer Centre London, London, UK; Clinical Adviser); Z Raytor (The Glen Hospital, Bristol, UK); B Richmond (St Mary's Hospital, London, UK; deceased); J Robertson (Nottingham City Hospital, Nottingham, UK); R Sainsbury (The London Breast Clinic, London, UK); P Sauven (Broomfield NHS Trust, Chelmsford, UK); R J Simes (University of Sydney, Sydney, Australia); R Stewart (Kettering General Hospital, Kettering, UK); A Stotter (University Hospital of Leicester, Leicester, UK); A Thompson (MD Anderson, Houston, Texas, united States); J Toy (Cancer Research UK, London, UK; Observer); P Twentyman (UKCCCR, London, UK; Observer, deceased); C Underhill (Border Medical Oncology, Wodonga, Australia); R Ward (St Vincent's Hospital, Sydney, Australia); S White (Austin Health, Heidelberg, Australia); A Wilkinson (Belfast City Hospital, Belfast, UK); S Wilkinson (Royal Hobart Hospital, Hobart, Australia); J Williamson (City Hospital NHS Trust, Birmingham, UK); C Wynne (Christchurch Hospital, Christchurch, New Zealand).

IBIS Coordinating Centre—Cancer Research UK, London, UK: R Edwards and R Kealy.

IBIS-ANZ BCTG Operations Office—V Gebski, D Lindsay, A Melmeth, A Newton, L Paksec, M Seccombe, R Thornton.

Supplementary Table 1: Number of women recruited per centre in IBIS-I.

Centre	Number of women recruited
BRISTOL (UK)	872
MELBOURNE(ANZ)	691
GUYS (UK)	557
MANCHESTER (UK)	524
PERTH(ANZ)	476
SOUTHAMPTON (UK)	400
SYDNEY(ANZ)	384
EDINBURGH (UK)	256
CARDIFF (UK)	249
BRISBANE(ANZ)	244
NEWCASTLE(ANZ)	242
CHELMSFORD (UK)	209
ABERDEEN (UK)	177
ADELAIDE(ANZ)	176
NEWZEALAND(ANZ)	161
HOBART(ANZ)	159
CANBERRA(ANZ)	143
FINLAND (FN)	135
GLASGOW (UK)	127
NEWCASTLE (UK)	122
LEEDS (UK)	118
HUDDERSFIELD (UK)	114
BIRMINGHAM (UK)	103
CAMBRIDGE (UK)	94
NOTTINGHAM (UK)	84
BELFAST (UK)	84
DUNDEE (UK)	49
TICINO (CH)	46
LEICESTER (UK)	44
UC HOSPITAL (UK)	32
OXFORD (UK)	32
LIVERPOOL (UK)	17
KETTERING (UK)	13
BELGIUM (BE)	9
ST GALLEN (CH)	8
ESPANA (ES)	2
MONAGHAN (IR)	1

Supplementary Table 2: Baseline demographics according to treatment allocation.

	Placebo (N=3575)	Tamoxifen (N=3579)
**Median age (years), (IQR)**	49·9 (46·1 to 55·0)	49·9 (45·9 to 55·0)
**Median BMI (kg/cm^2), (IQR)**	26·1 (23·2 to 29·6)	26·0 (23·3 to 29·7)
**MHT use**		
During trial only	1414 (49·5%)	1462 (40·9%)
Before trial only	380 (10·6%)	399 (11·2%)
Never	1769 (49·5%)	1708 (47·7%)
**Hysterectomy (%)**	1283 (35.9%)	1232 (34.4%)

*IQR=Interquartile Range, BMI=Body Mass Index, MHT=Menopausal Hormone Therapy

Supplementary Table 3: Thromboembolic, cardiovascular, and cerebrovascular events according to treatment allocation.

	Placebo	Tamoxifen	OR (95% CI)
**Thromboembolic events**			
DVT	29	50	1·73 (1·07-2·85)
PE	22	30	1·37 (0·76-2·49)
Superficial thrombophlebitis	11	24	2·19 (1·03-4·95)
**All**	**62**	**104**	**1·70 (1·22-2·37)**
**Cardiovascular events**			
Myocardial infarction	17	13	0·76 (0·34-1·67)
Angina	51	60	1·18 (0·80-1·75)
**All**	**153**	**141**	**0·92 (0·72-1·17)**
**Cerebrovascular events**			
Stroke/CVA	28	30	1·07 (0·62-1·86)
TIA	40	27	0·67 (0·40-1·12)
**All**	**74**	**62**	**0·83 (0·58-1·19)**

*DVT=Deep vein thrombosis, PE=Pulmonary embolism, CVA=Cerebrovascular accident, TIA = Transient ischaemic attack

Supplementary Figure 1: Forest plot for invasive breast cancer characteristics according to follow-up period (red: 0-10 years, blue: 10+ years).

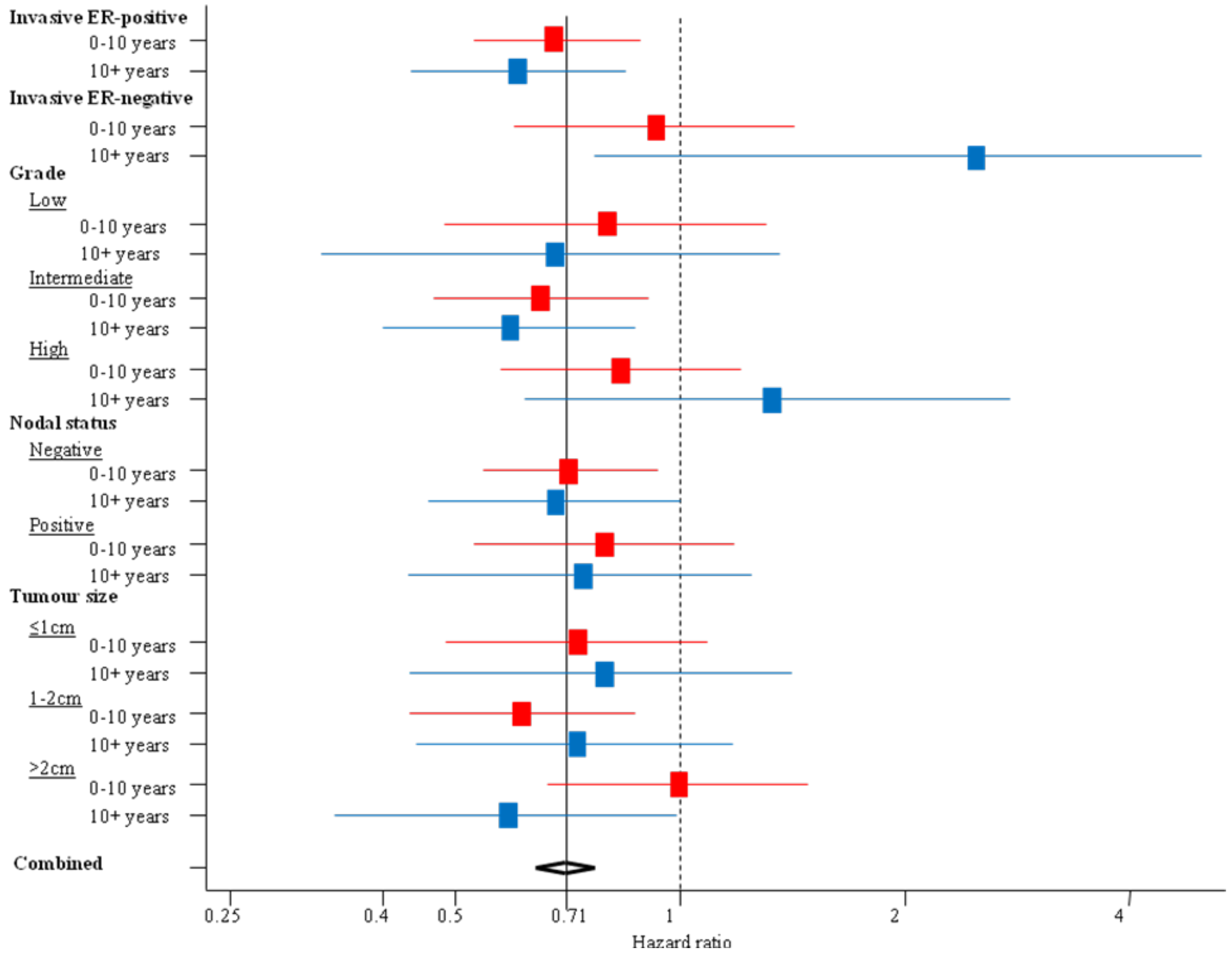

Supplement: Supplementary appendix [file mmc1.pdf]
